# Impact of catheter contact angle on lesion formation and durability of pulmonary vein isolation

**DOI:** 10.1007/s10840-022-01131-1

**Published:** 2022-01-26

**Authors:** Masayuki Ohta, Kentaro Hayashi, Hiroyuki Sato, Takahiro Noto, Kandoh Kawahatsu, Masaya Katagiri, Tomohiro Mita, Yoshio Kazuno, Shunsuke Sasaki, Takahiro Doi, Mitsugu Hirokami, Satoshi Yuda

**Affiliations:** 1 Department of Cardiology, Teine Keijinkai Hospital, Sapporo, Hokkaido Japan; 2 Department of Cardiology, Ageo Central Medical Hospital, Ageo, Saitama Japan

**Keywords:** Atrial fibrillation, Catheter ablation, Pulmonary vein isolation, Catheter contact angle, Perpendicular contact

## Abstract

**Purpose:**

This study is aimed to evaluate the impact of catheter contact angle on lesion formation and durability of pulmonary vein isolation (PVI).

**Methods:**

Both in vitro experiment and retrospective observational study were conducted. For in vitro experiment, radiofrequency lesions were created on explanted swine hearts in three different catheter contact angles (0°, 45°, and 90°). In the retrospective observational study, we assessed patients who had undergone repeat catheter ablation due to atrial fibrillation recurrence after initial PVI. When pulmonary vein (PV) reconnection was observed, we analyzed the previous ablation points within and without the gap area. The gap areas were where ablation had changed the PV activation sequence or eliminated the PV potential in the repeat session.

**Results:**

In the in vitro experiment, lesion width was the smallest (5.3 ± 0.4 mm) in perpendicular contact compared to 0° (vs 5.8 ± 0.5 mm, *p* = 0.040) and 45° (vs 6.4 ± 0.4 mm, *p* < 0.001). In the retrospective observational study, we assessed 666 tags of 16 patients with PV reconnections, and 60 tags were in the gap area. Tags in the gap area had longer interlesion distance (odds ratio [*OR*] 1.49, *p* < 0.001), greater contact force variability (*OR* 1.03, *p* = 0.008), and higher rate of perpendicular contact (*OR* 3.26, *p* < 0.001) on multivariate analysis.

**Conclusions:**

Perpendicular contact was associated with a smaller lesion and higher rate of PV reconnection.

## Introduction

Pulmonary vein isolation (PVI) is a cornerstone therapy in catheter ablation of atrial fibrillation (AF) [1]. Studies on AF recurrence rates after initial ablation procedures have been variable, ranging from 20 to 80% in several studies, and 20–50% of patients require a repeat ablation procedure to achieve sinus rhythm during long-term follow-up [2, 3]. The left atrial (LA)–pulmonary vein (PV) reconnection after initial PVI is one of the main causes of AF recurrence [4]. Therefore, durable lesion formation is important in preventing recurrences of AF.

Three-dimensional mapping systems, irrigation catheters, and contact-force (CF) sensing catheter have been developed to improve lesion durability. Furthermore, force–time integral (FTI) and other lesion indexing algorithms, including ablation index (AI), have been used as surrogates for radiofrequency (RF) dose and lesion size. However, LA–PV reconnection remains unclear with these instruments [5–7].

Catheter stability can be a factor of durable lesion formation beyond FTI or AI. Makimoto et al. supposed that, even if the mean CF applied is within the target range, a large variability of the actual CF value during RF application suggests catheter instability [8]. Ullah et al. have also shown larger CF variability resulting in smaller impedance drop [9]. Moreover, they showed several factors associated with lesser impedance drop: catheter CF variability > 5 g, catheter drift > 3.5 mm, sinus rhythm, and perpendicular contact [9]. However, little is known about the effect of catheter contact angle against the myocardium on lesion formation and durability of PVI.

This study aimed to evaluate the impact of catheter contact angle on lesion formation and durability of PVI.

## Methods

We conducted both in vitro experiment and retrospective observational study. The retrospective analysis was in accordance with the “Declaration of Helsinki” and approved by the Ethics Committee of Teine Keijinkai Hospital (2–020,132-00).

### In vitro experiments

**Fig. 1:**
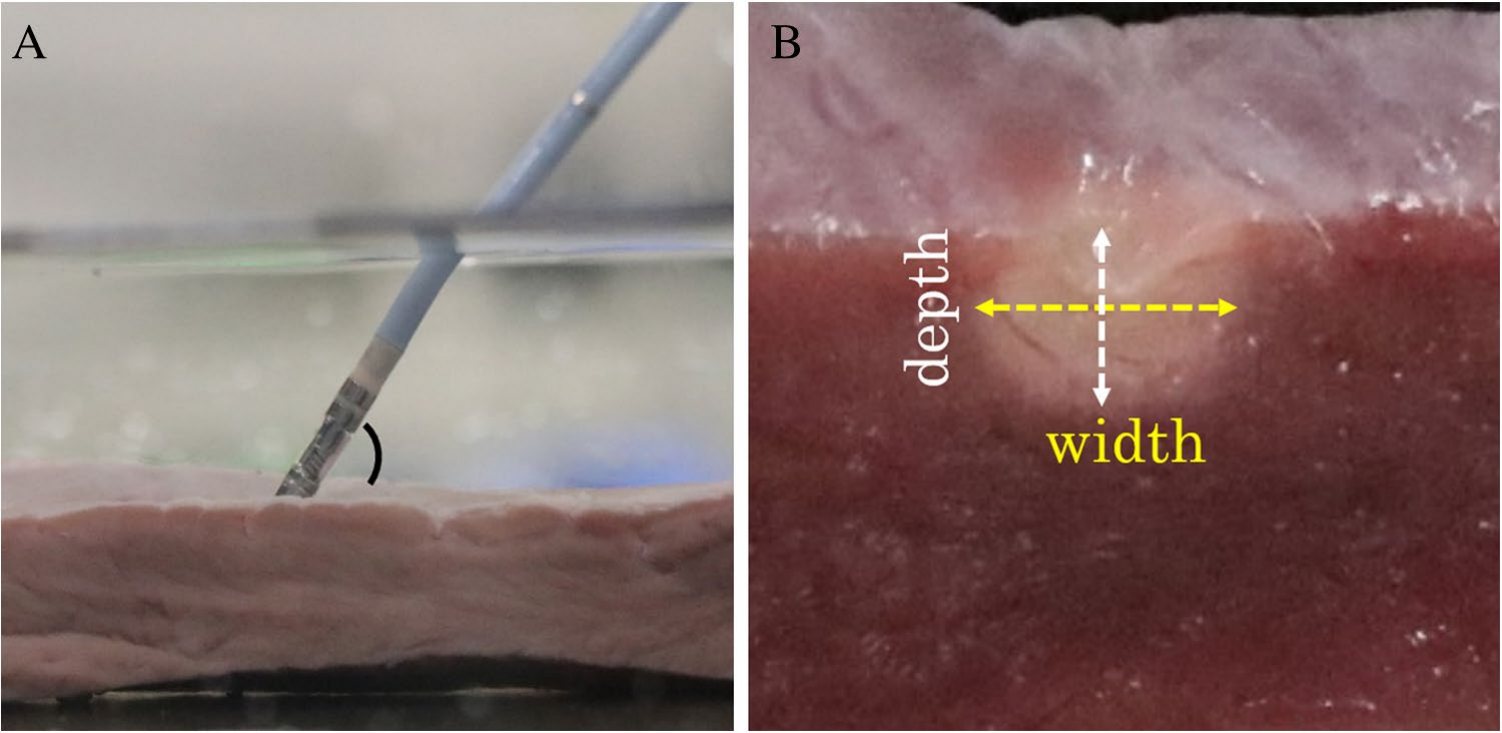
Representative catheter position during radiofrequency application to Swine’s heart (**A**) and the lesion (**B**). Radiofrequency applications were conducted on the swine’s heart ventricular tissue in a saline bath (**A**). The lesions were sectioned along their major axis, and widths and depths were measured (**B**)

The swine’s heart ventricular tissue was dissected and pinned to a tissue holder that was fixed in a saline bath. Using a CARTO system (Biosense Webster, Inc.) and a ThermoCool SmartTouch irrigated-tip CF sensing RF ablation catheter (Biosense Webster, Inc.), RF lesions were created with ablation power of 20 W for 15 s with saline 8-mL/min irrigation in two different catheter CFs (5 g and 10 g) and three different catheter contact angles (0°, 45°, and 90°) (Fig. 1A). Five radiofrequency applications were applied under each condition, and 30 lesions were created. The lesions were sectioned along their major axis, and widths and depths were measured (Fig. 1B). We also investigated each FTI, AI, impedance before RF application (pre-impedance), impedance after RF application (post-impedance), and impedance change (impedance drop).

### Retrospective observation study

#### Patient population

We included 27 consecutive patients who underwent repeat catheter ablation due to AF recurrence after initial PVI using the CARTO system and VisiTag™ module from October 1, 2017, to August 31, 2019. All patients provided written informed consent before the procedures.

#### Catheter ablation procedure

**Fig. 2:**
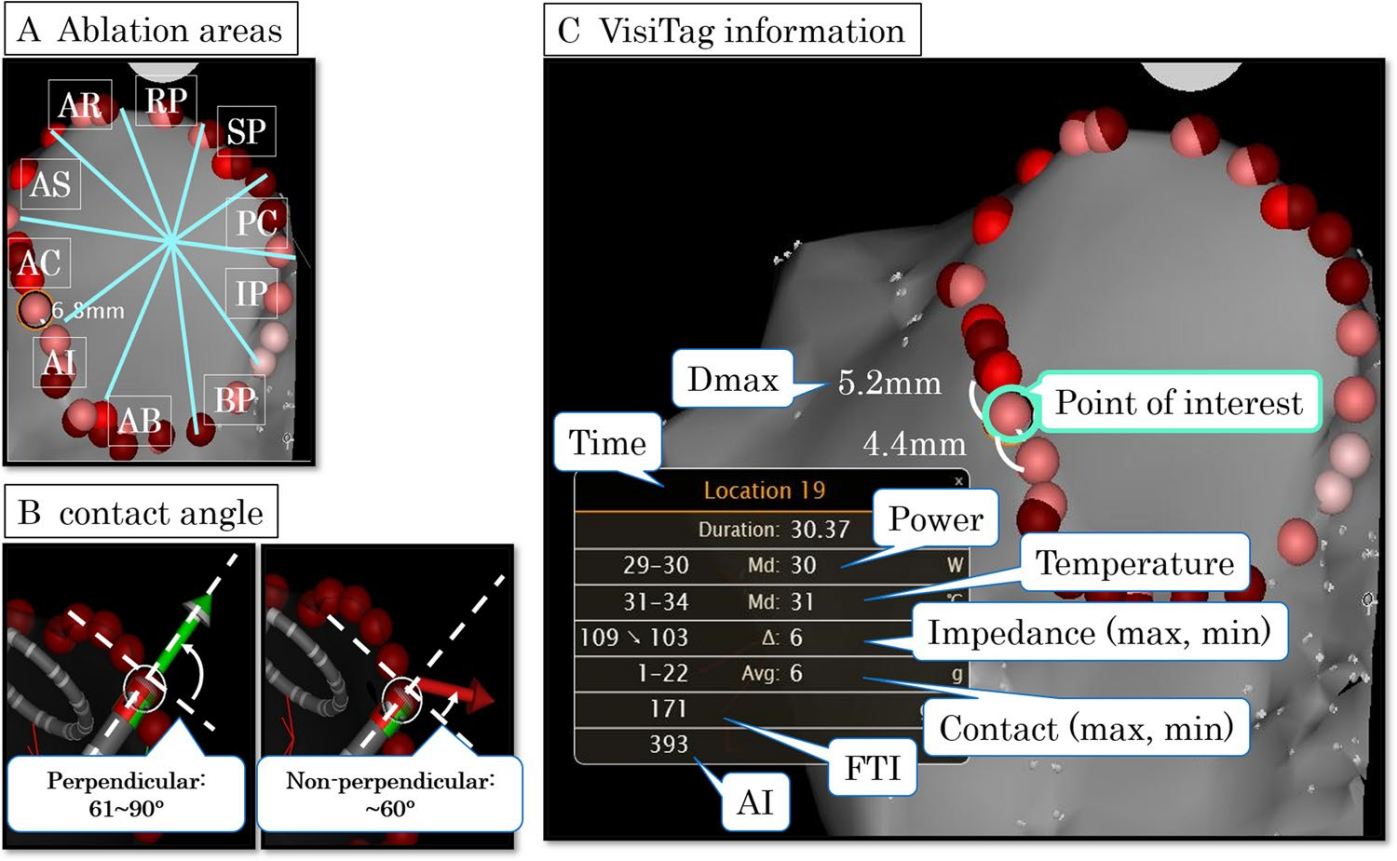
Representative VisiTags shown in the CARTO system. Ablation areas were divided into ten areas for each ipsilateral pulmonary vein (**A**). Ablation points were manually tagged to record the catheter position and the vector arrows showing actual angle of catheter tip to tissue. Perpendicular contact was defined when the angle between the vertical line to the ablation catheter and the vector arrow was > 60° (**B**). Recorded VisiTag information (**C**). Dmax is the maximum distance to the adjacent tags. AR, anterior roof; AS, anterior superior; AC, anterior carina; AI, anterior inferior; AB, anterior bottom; PR, posterior roof; PS, posterior superior; PC, posterior carina; PI, posterior inferior; PB, posterior bottom; FTI, force–time integral; AI, ablation index

All patients underwent the procedure under sedation. Transesophageal echocardiography was performed to rule out thrombus in the left atrium before the procedure. After dual transseptal puncture, 3D electroanatomical maps of the LA and PVs were reconstructed with a PentaRay (Biosense Webster, Inc.) in each session. In the first session, extensive encircling PVI was performed by point-by-point RF application using a ThermoCool SmartTouch irrigated-tip CF sensing RF ablation catheter (Biosense Webster, Inc.). Ablation power was 20–25 W for posterior wall and 25–30 W for anterior wall, and the target ablation index was 375 for posterior wall and 425 for anterior wall. Ablation lesion was manually tagged routinely after the catheter was stabilized to record the catheter position and the vector arrows showing actual angle of catheter tip to the tissue (Fig. 2B). VisiTag™ was also annotated automatically on the lesion, based on the location stability setting of 3 mm for 10 s with respiration adjustment and without force over time or impedance drop filters. When first-pass isolation was not achieved, reablation would be applied at the sites with conduction gap until both exit and entrance blocks were achieved. In the repeat session, RF ablation was applied at the sites with conduction gap if LA–PV reconnection was observed.

#### Study measurements

We assessed the previous RF application of patients with LA–PV reconnection confirmed in the repeat session. The tags were excluded from the analysis if two or more VisiTags were annotated with one RF application. The PVI line of each ipsilateral PV was divided into ten areas by anterior/posterior and roof/superior/carina/inferior/bottom (Fig. 2A). The gap areas were defined where RF ablation had changed the PV activation sequence or eliminated the PV potential in the repeat session. The angle between the vector arrow and vertical line to the ablation catheter was measured by a semicircular protractor, using the ablation point recorded in the initial session. Perpendicular contact was defined when the angle between the vertical line to the ablation catheter and the vector arrow was > 60° (Fig. 2B). We investigated all VisiTag™ information of initial PVI: ablation time, ablation power, catheter temperature, CF, FTI, AI, generator impedance, and maximum distance to the adjacent tags (Dmax) (Fig. 2C). CF variability was defined as the difference between maximum and minimum CF.

### Statistical analysis

In *in vitro* experiments, to compare three different catheter contact angles, we performed the one-way analysis of variance. We also performed Student’s *t*-test to compare two groups. In the retrospective analysis of PVI, all ablation information was compared using Student’s *t*-test and chi-square test for continuous and categorical variables, respectively. Univariate analysis was conducted on factors identified as significant by these methods; then, multivariate analysis with a logistic regression model was performed using the significant variables identified by univariate analysis to define an independent predictor of the gap area. We also assessed the points without Dmax > 6 mm and evaluated them in the same way. Receiver operating characteristic analysis was performed to determine the optimal cut-off value of Dmax that exhibited optimal sensitivity and specificity. The value for the maximum Youden index was considered as the optimal cut-off point. A *P*-value < 0.05 was considered statistically significant. All statistical analyses were performed using EZR (Saitama Medical Center, Jichi Medical University, Saitama, Japan), a graphical user interface for R (The R Foundation for Statistical Computing, Vienna, Austria) designed to add statistical functions frequently used in biostatistics [10].

## Results

### In vitro experiments

**Fig. 3:**
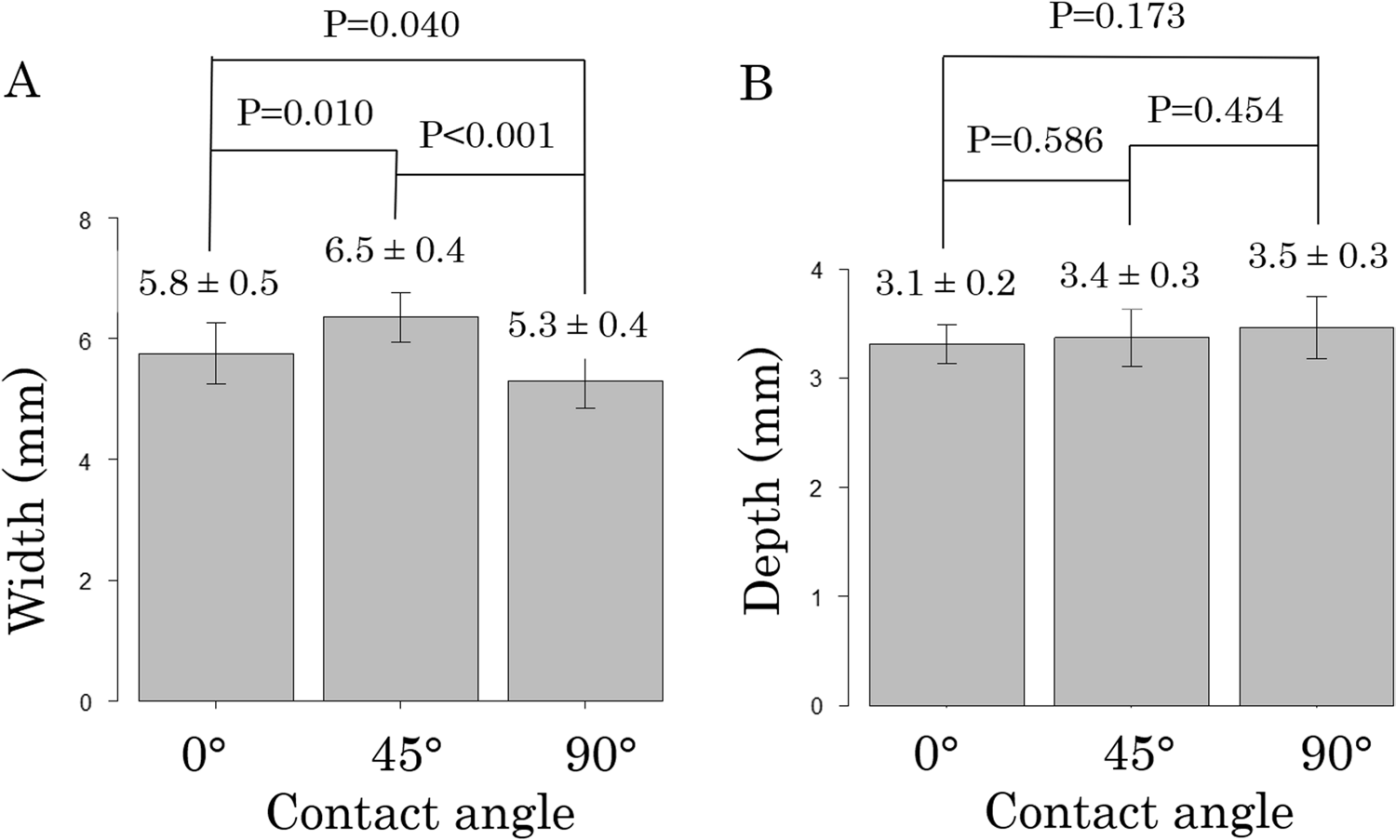
Lesion widths (**A**) and depths (**B**) under different catheter contact angles. The widths (**A**) and depths (**B**) of lesions created during in vitro experiment were shown. *P*-values were generated using Student’s *t*-test

In perpendicular contact (contact angle of 90°), lesion width was the smallest (5.3 ± 0.4 mm) compared to 0° (vs 5.8 ± 0.5 mm, *p* = 0.040) and 45° (vs 6.4 ± 0.4 mm, *p* < 0.001), while lesion depth had no significant differences (Fig. 3). FTI and AI had no significant differences among the three groups (Table 1). As for generator impedance, both impedance drop and impedance before RF application were the largest in 45° (Table 1).

**Table 1:** Ablation index (AI), force–time integral (FTI), and impedance information under different catheter contact angles

	0° *n* = 10	45° *n* = 10	90° *n* = 10	*P*-value
AI, mean (± SD)	292.20 (28.48)	295.80 (23.22)	299.70 (25.51)	0.811
FTI, g•s, mean (± SD)	216.40 (84.70)	226.80 (72.43)	239.70 (80.25)	0.807
Pre-impedance, Ω, mean (± SD)	136.20 (12.04)	147.10 (5.70)	134.00 (5.46)	0.003
Post-impedance, Ω, mean (± SD)	128.10 (10.14)	127.30 (2.91)	122.40 (3.72)	0.121
Impedance drop, Ω, mean (± SD)	8.10 (3.48)	19.80 (4.89)	11.60 (2.59)	< 0.001

*P*-values were generated using one-way analysis of variance. *AI*, ablation index; *FTI*, force–time integral

### Retrospective analysis of PVI

Table 2 shows the baseline characteristics of the included patients. The overall patients included 22 men (81.5%) with mean age of 60.7 ± 9.4 years. Ten patients (37.0%) had paroxysmal AF. The mean period from the previous session was 358.4 ± 202.4 days. The LA–PV reconnections were detected in 21 ipsilateral PVs (38.9%) in 16 patients (59.3%). We assessed 666 tags of these 16 patients, and 60 tags were in the gap area. The left anterior carina area was the most frequent site of the gap area (*n* = 14), following the right posterior carina area (*n* = 11), the right posterior superior area (*n* = 8), and the left posterior superior area (*n* = 5). The tags in the gap area had higher rate of perpendicular contact (50.0% vs 16.7%, *p* < 0.001), longer Dmax (6.9 ± 1.8 vs 5.9 ± 1.5, *p* < 0.001), lower minimum CF (2.3 ± 3.5 vs 4.8 ± 5.1, *p* < 0.001), greater CF variability (30.2 ± 20.9 vs 24.1 ± 14.5, *p* = 0.003), lower FTI (243.2 ± 142.0 vs 289.0 ± 145.8, *p* = 0.020), higher ablation power, and lower impedance (maximum, minimum), although other variables, such as AI and impedance drop, showed no significant differences (Table 3). In the multivariate analysis, longer Dmax (*OR* 1.49, *p* < 0.001), larger CF variability (*OR* 1.03, *p* = 0.008), and perpendicular contact (*OR* 3.26, *p* < 0.001) were associated with the gap area (Table 3). Moreover, 19 of 381 points with Dmax ≤ 6 mm were detected in the gap area. In ablation points with Dmax ≤ 6 mm, larger CF variability (*OR* 1.05, *p* = 0.005) and perpendicular contact (*OR* 3.47, *p* = 0.025) were the predictors of PV reconnection on multivariate analysis (Table 4).

**Table 2:** Baseline characteristics of the included patients

Baseline characteristics	Overall *N* = 27	Gap ( −) *N* = 11	Gap ( +) *N* = 16	*P*-value
Age	60.7 (9.4)	58.9 (7.5)	62.0 (10.6)	0.411
Sex				
Male, *n* (%)	22 (81.5)	9 (81.8)	13 (81.2)	1.000
Female, *n* (%)	5 (18.5)	2 (18.2)	3 (18.8)	
Type of atrial fibrillation				
Paroxysmal, *n* (%)	10 (37.0)	3 (27.3)	7 (43.8)	0.448
Persistent, *n* (%)	17 (63.0)	8 (72.7)	9 (56.2)	
Body mass index, kg/m^2^, mean (± SD)	1.8 (0.2)	1.9 (0.2)	1.8 (0.2)	0.217
Congestive heart failure, *n* (%)	8 (29.6)	5 (45.5)	3 (18.8)	0.206
Diabetes mellitus, *n* (%)	4 (14.8)	4 (36.4)	0 (0.0)	0.019
Hypertension, *n* (%)	17 (63.0)	7 (63.6)	10 (62.5)	1.000
Stroke, *n* (%)	4 (14.8)	2 (18.2)	2 (12.5)	1.000
Coronary artery disease, *n* (%)	1 ( 3.7)	0 (0.0)	1 (6.2)	1.000
CHADS_2_ score, mean (± SD)	1.4 (1.3)	1.8 (1.2)	1.2 (1.4)	0.226
CHA_2_DS_2_-VASc score, mean (± SD)	2.0 (1.7)	2.3 (1.6)	1.8 (1.8)	0.449
Left atrial diameter, mm, mean (± SD)	42.8 (8.3)	45.7 (8.1)	40.8 (8.1)	0.133
Left atrial volume, mL, mean (± SD)	81.9 (28.7)	86.7 (33.4)	78.4 (25.3)	0.480
First-pass isolation, *n* (%)	13 (48.1)	6 (54.5)	7 (43.8)	0.688

CHADS_2_ = congestive heart failure, 1 point; hypertension, 1 point; ≥ 75 years, 2 points; diabetes mellitus, 1 point; previous stroke, transient ischemic attack or thromboembolism, 2 points

CHA2DS2-VASc = congestive heart failure, 1 point; hypertension, 1 point; ≥ 75 years, 2 points; diabetes mellitus, 1 point; previous stroke, transient ischemic attack or thromboembolism, 2 points; vascular disease, 1 point; 65–74 years, 1 point; female sex, 1 point

**Table 3:** Comparison of radiofrequency ablation information of previous pulmonary vein isolation points between gap and no-gap area

	Gap ( −)	Gap ( +)	*P*-value	*OR* (95% *CI*)	*P*-value
	*N* = 606	*N* = 60			
Temperature, °C mean (± SD)	33.3 (2.7)	33.1 (1.9)	0.523		
Time, s, mean (± SD)	22.7 (6.4)	23.4 (9.0)	0.433		
Power, W, mean (± SD)	25.4 (3.8)	26.5 (4.1)	0.029	1.08 (1.00–1.18)	0.056
Dmax, mm, mean (± SD)	5.9 (1.5)	6.9 (1.8)	< 0.001	1.49 (1.26–1.76)	< 0.001
Maximum CF, g, mean (± SD)	28.9 (15.9)	32.5 (21.1)	0.113		
Minimum CF, g, mean (± SD)	4.8 (5.1)	2.3 (3.5)	< 0.001	0.92 (0.81–1.04)	0.170
CF variability, g, mean (± SD)	24.1 (14.5)	30.2 (20.9)	0.003	1.03 (1.01–1.05)	0.008
FTI, g•s, mean (± SD)	289.0 (145.8)	243.2 (142.0)	0.020	1.00 (1.00–1.00)	0.296
AI, mean (± SD)	376.9 (56.2)	376.1 (59.7)	0.915		
Maximum impedance, Ω, mean (± SD)	120.5 (12.4)	115.3 (12.7)	0.002	0.99 (0.93–1.05)	0.748
Minimum impedance, Ω, mean (± SD)	110.9 (10.9)	106.3 (11.1)	0.008	0.99 (0.93–1.06)	0.761
Impedance drop, Ω, mean (± SD)	10.3 (5.7)	8.9 (4.5)	0.058		
Contact angle, *n* (%)					
Non-perpendicular	505 (83.3)	30 (50.0)		―	―
Perpendicular	101 (16.7)	30 (50.0)	< 0.001	3.26 (1.71–6.22)	< 0.001

*P*-values were generated using Student’s *t*-test and chi-square test for continuous and categorical variables, respectively. Then, multivariate analysis with a logistic regression model was performed using the significant variables identified by univariate analysis to define an independent predictor of the gap area. Dmax is the maximum distance to the adjacent tags. *CF*, contact force; *FTI*, force–time integral; *AI*, ablation index

**Table 4:** Comparison of radiofrequency ablation information of previous pulmonary vein isolation points with maximum distance to the adjacent tags ≤ 6 mm between gap and no-gap area

	Gap ( −)	Gap ( +)	*P*-value	*OR* (95% *CI*)	*P*-value
	*N* = 362	*N* = 19			
Temperature, °C, mean (± SD)	33.1 (2.8)	32.2 (1.3)	0.149		
Time, s, mean (± SD)	22.2 (5.9)	21.5 (5.0)	0.621		
Power, W, mean (± SD)	25.4 (3.9)	26.8 (3.4)	0.104		
Dmax, mm, mean (± SD)	5.0 (0.8)	5.0 (0.7)	0.897		
Maximum CF, g, mean (± SD)	29.6 (16.1)	36.1 (14.8)	0.083		
Minimum CF, g, mean (± SD)	4.7 (5.3)	1.8 (2.0)	0.019	0.94 (0.73–1.20)	0.598
CF variability, g, mean (± SD)	24.8 (14.8)	34.3 (14.5)	0.007	1.05 (1.01–1.08)	0.005
FTI, g•s, mean (± SD)	283.5 (144.3)	214.8 (80.5)	0.041	0.99 (0.99–1.00)	0.066
AI, mean (± SD)	373.0 (55.3)	370.7 (40.5)	0.861		
Maximum impedance, Ω, mean (± SD)	120.2 (12.3)	117.1 (10.8)	0.276		
Minimum impedance, Ω, mean (± SD)	110.1 (11.0)	108.4 (10.0)	0.507		
Impedance drop, Ω, mean (± SD)	10.2 (5.6)	8.4 (4.8)	0.168		
Contact angle, *n* (%)					
Non-perpendicular	294 (81.2)	7 (36.8)		―	―
Perpendicular	68 (18.8)	12 (63.2)		3.47 (1.17–10.2)	0.025

*P*-values were generated using Student’s *t*-test and chi-square test for continuous and categorical variables, respectively. Then, multivariate analysis with a logistic regression model was performed using the significant variables identified by univariate analysis to define an independent predictor of the gap area. Dmax is the maximum distance to the adjacent tags. *CF*, contact force; *FTI*, force–time integral; *AI*, ablation index

**Fig. 4:**
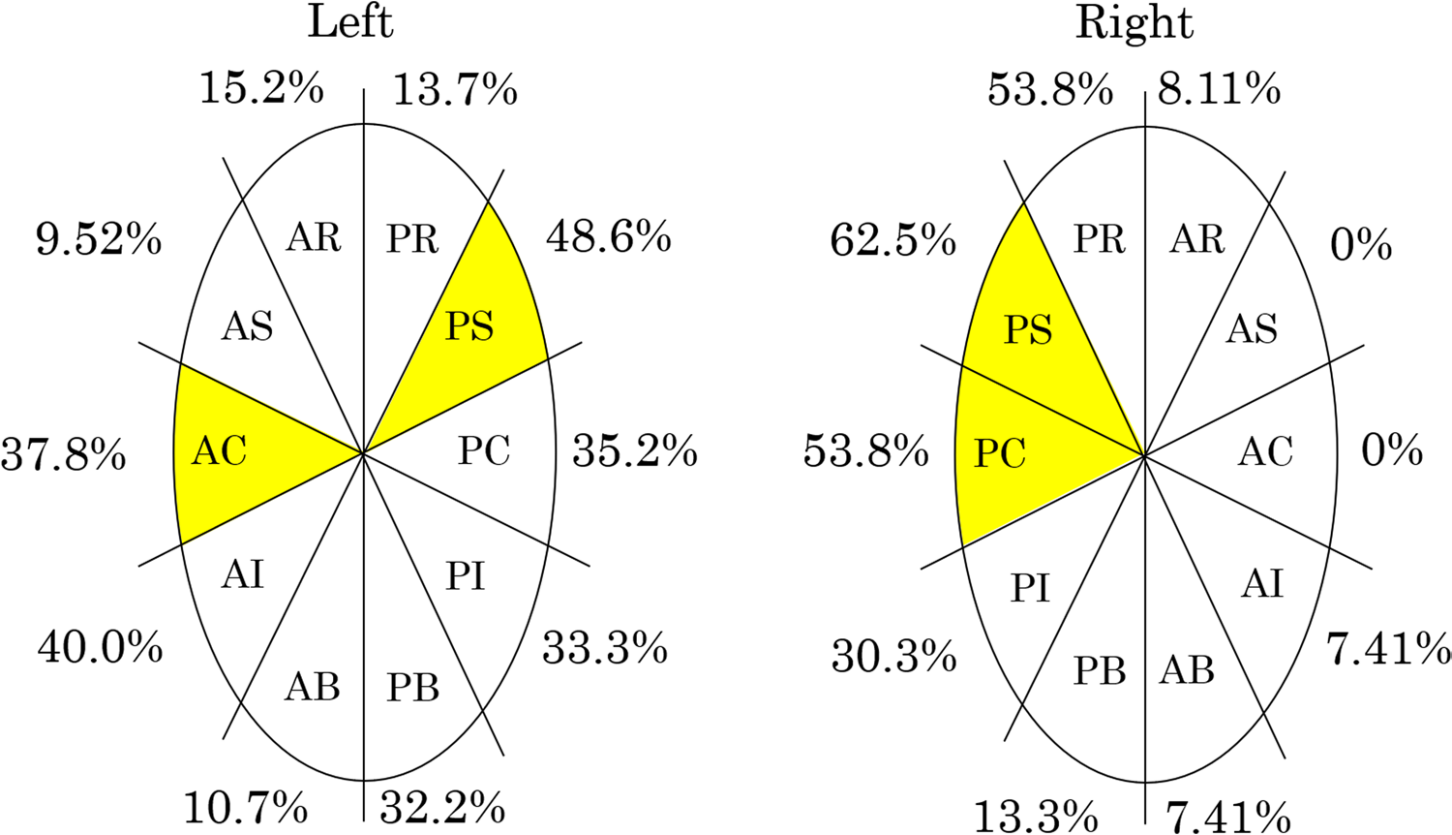
The common gap areas and the frequency of perpendicular contact in each area. The common gap areas are highlighted, and the frequency of perpendicular contact is shown in each area. The most common gap area is the left carina anterior area (*n* = 14), following the right carina posterior area (*n* = 11), the right posterior superior area (*n* = 8), and the left posterior superior area (*n* = 5). AR, anterior roof; AS, anterior superior; AC, anterior carina; AI, anterior inferior; AB, anterior bottom; PR, posterior roof; PS, posterior superior; PC, posterior carina; PI, posterior inferior; PB, posterior bottom

Table 5 shows the comparison between non-perpendicular (*n* = 535) and perpendicular contact (*n* = 131). Although maximum CF was significantly high in perpendicular contact than non-perpendicular contact (31.9 ± 21.9 vs 28.6 ± 14.8, *p* < 0.040), minimum CF was low (2.2 ± 3.4 vs 5.2 ± 5.2, *p* < 0.001), resulting in larger CF variability (29.8 ± 20.9 vs 23.4 ± 13.2, *p* < 0.001). As for generator impedance, perpendicular contact was associated with lower maximum and minimum impedance with lower impedance drop compared to non-perpendicular contact. Figure 4 shows the common gap areas and the frequency of perpendicular contact in each area. The common gap areas were left anterior carina, left posterior-superior area, right postero-superior area and right posterior carina. The frequency of perpendicular contact was 37.8–65.2%.

**Table 5:** Comparison of radiofrequency ablation information of previous pulmonary vein isolation points between parallel and perpendicular contact

	Non-perpendicular	Perpendicular	*P*-value
	*N* = 535	*N* = 131	
Tags within the gap area, *n*, (%)	30 (5.6)	30 (23.0)	< 0.001
Temperature, °C, mean (± SD)	33.4 (2.7)	33.1 (2.0)	0.279
Time, s, mean (± SD)	22.7 (6.5)	23.0 (7.2)	0.657
Power, W, mean (± SD)	25.5 (3.8)	25.7 (4.0)	0.563
Dmax, mm, mean (± SD)	6.0 (1.4)	5.9 (1.8)	0.335
Maximum CF, g, mean (± SD)	28.6 (14.8)	31.9 (21.9)	0.040
Minimum CF, g, mean (± SD)	5.2 (5.2)	2.2 (3.4)	< 0.001
CF variability, g, mean (± SD)	23.4 (13.2)	29.8 (20.9)	< 0.001
FTI, g•s, mean (± SD)	298.2 (145.0)	230.3 (137.2)	< 0.001
AI, mean (± SD)	380.8 (56.8)	360.6 (52.4)	< 0.001
Maximum impedance, Ω, mean (± SD)	121.1 (12.6)	115.6 (10.9)	< 0.001
Minimum impedance, Ω, mean (± SD)	110.4 (11.2)	107.3 (9.8)	0.004
Impedance drop, Ω, mean (± SD)	10.7 (5.8)	8.3 (4.4)	< 0.001

*P*-values were generated using Student’s *t*-test and chi-square test for continuous and categorical variables, respectively. Dmax is the maximum distance to the adjacent tags. *CF*, contact force; *FTI*, force–time integral; *AI*, ablation index

## Discussion

Our data shows that (I) the lesion size is small in perpendicular contact and (II) perpendicular contact is a strong predictor of the LA–PV reconnection in PVI.

### RF lesion created in different catheter contact angles

In our in vitro study, although AI and FTI did not show significant differences among different catheter contact angles, the lesion width was small in the perpendicular contact and large in 45°. We also found that initial impedance was significantly high in 45°.

Recent in vitro study demonstrated by V. Calzolari et al. [11] is consistent with our study. RF lesions created on a porcine heart with different contact angles were evaluated, and they measured both superficial lesion size and cross-sectional lesion size. They have shown that both superficial lesion length and cross-sectional lesion length were larger in parallel contact than in perpendicular contact.

In vitro experiments have shown that FTI or ablation index is known to be associated with lesion size [12, 13]. However, AI and FTI do not consider contact angle at ablation sites that affect initial impedance, which in turn can influence lesion formation [14].

Lesions formed by RF application consist of two regions: a central region formed by resistive heating and a surrounding hemorrhagic region formed by conductive heat transfer that depends on application time. The lesion size influenced by resistive heating is determined by the amount of current delivered to the tissue [15]. According to Joule’s law, the amount of current is inversely proportional to the circuit impedance. Impedance represents resistance to current flow in the local tissue if other variables are under the same condition, including the RF generator, catheter, tissue distant to the heart, and return electrode, and in the general circuit cabling [16]. The resistance of local tissue can be affected by tissue properties, catheter CF, or surface area covered by the catheter tip. In our study, as the catheter CF was fixed, lower impedance before ablation in perpendicular contact might be due to the narrower contact area between the catheter tip and myocardium, resulting in a small lesion.

### Factors of LA–PV reconnection in PVI

In our study, 38.9% of PVs (21 of 54) in 16 of 27 patients (59.3%) had PV reconnection in the repeat session. Univariate analysis showed several factors associated with PV reconnection: long interlesion distance; low minimum CF; high CF variability; low initial, maximum, and minimum impedance; and perpendicular contact.

The importance of catheter CF has been investigated in studies examining the reconnection of segments in PVI line during AF ablation. The EFFICAS I study has shown that PV reconnection at 3 months after PVI was strongly associated with minimum CF and minimum FTI at the site of gap. In the EFFICAS II study, although the PV reconnection rates decreased following the CF guidance from EFFICAS I (target 20 g, range 10–30 g, minimum 400 g*s FTI), 15% of PV reconnections were still detected [6].

Catheter stability has been considered an important factor beyond FTI. Catheter stability was previously assessed using CF variability or relative standard deviation of CF. One in vitro study using a contractile bench model simulating the beating heart showed more variable contact resulting in smaller lesions [12]. In the clinical study, Ullah et al. [9] demonstrated that lesions delivered with variability over 5 g are associated with a lower impedance drop, despite delivering the same FTI. They suggested that high degree of CF variability leads to a cooling effect on the tissue through stretching and unstretching, reducing the effect of ablation. Makimoto et al. [8] demonstrated that the relationship between the average and standard deviation of CF is correlated with acute PV reconnection. They show that ablation segments along the circumferential ablation lines delivered with lower average force and high variability are prone to form reconnection gaps. In our study, compared to non-perpendicular contact, larger maximum CF, lower minimum CF, and larger CF variability during application were observed especially in perpendicular contact, indicating larger CF variability. Therefore, perpendicular contact can be a predictive factor of catheter instability leading to a nondurable lesion.

Interlesion distance is another important factor. A ‘CLOSE’ protocol ablation, targeting an interlesion distance ≤ 6 mm, AI ≥ 400, and ≥ 550 at the posterior and anterior walls, has been reported with better outcome with lower PV reconnection and lower AF recurrence rate [7]. In our study, 19 of 381 points with Dmax ≤ 6 mm were still detected in the gap area. Once interlesion distance ≤ 6 mm was achieved, CF variability and perpendicular contact were the only two predictors of PV reconnection in the multivariate analysis. Although it has been reported that CF variability did not differ if AI is constant [7], when focusing on patients with PV reconnection, CF variability and perpendicular contact might be additional factors beyond AI in terms of durable lesion formation.

### Difference between paroxysmal and persistent AF

When we compared paroxysmal AF (*n* = 10) with persistent AF (*n* = 17), the rate of perpendicular catheter contact was more frequently observed in paroxysmal AF (28.1% vs 6.7%, *p* = 0.001). Smaller left atrial volume was also observed in paroxysmal atrial fibrillation (60.1 ml vs 95.5 ml, *p* = 0.001). Therefore, because the catheter manipulation is limited in a small left atrium, achieving parallel catheter contact might be difficult in paroxysmal atrial fibrillation.

### Limitations

Several limitations need to be underlined. An in vitro study was conducted under limited setting of CF, ablation time, and power. To avoid steam pop phenomenon, lower CF and lower power were selected compared with other in vitro studies [11–14]. CF variability was not evaluated, and it is not clear whether the lesion size difference of in vitro study is clinically significant. In addition, the superficial lesion width was not measured because we measured the lesion size after lesions were sectioned along their major axis. The major limitation of the retrospective study was that it was conducted in a single-center and had a small sample size. In particular, most of the study population were men (81.5%), although men account for a large percentage (64–83%) in other studies of patients with atrial fibrillation recurrence after catheter ablation [17–19]. Moreover, the operator’s habit to apply the catheter might have effect on the gap area because the contact angle tended to be the same in the same area. Further research including an in vivo animal study and a prospective study at multiple facilities is required.

## Conclusion

Perpendicular contact was associated with smaller in vitro lesion size, and clinically, in this retrospective series including 27 patients, it was an indicator of catheter instability during PVI, resulting in nondurable lesion.

## Abbreviations

AF: Atrial fibrillation
AI: Ablation index
CF: Contact-force
EACTS: European Association of Cardio-Thoracic Surgery
FTI: Force–time integral
LA: Left atrial
PV: Pulmonary vein
PVI: Pulmonary vein isolation
ROC: Receiver operating characteristic curve
